# The immunological and prognostic significance of the diabetes mellitus-related gene WFS1 in endometrial cancer

**DOI:** 10.3389/fimmu.2024.1464421

**Published:** 2024-10-16

**Authors:** Wenzhe Li, Da Ke, Yi Xu, Ya Wang, Qian Wang, Jie Tan, Hongyan Wu, Xianglin Cheng

**Affiliations:** ^1^ Department of Endocrinology, The First Affiliated Hospital of Yangtze University, Jingzhou First People’s Hospital, Jingzhou, Hubei, China; ^2^ Department of Hubei Provincial Clinical Research Center for Personalized Diagnosis and Treatment of Cancer, The First Affiliated Hospital of Yangtze University, Jingzhou First People’s Hospital, Jingzhou, Hubei, China; ^3^ Department of Hematology, The First Affiliated Hospital of Yangtze University, Jingzhou First People’s Hospital, Jingzhou, Hubei, China

**Keywords:** WFS1, endometrial cancer, molecular typing, diabetes mellitus, prognostic markers

## Abstract

**Background:**

Diabetes is associated with the incidence and prognosis of various malignancies, most notably endometrial cancer (EC). This study investigated the connection between diabetes and EC, with a specific focus on elucidating the biological implications of the diabetes mellitus (DM)-related gene WFS1.

**Methods:**

Using the CTD, GeneCards, and GSEA databases, we identified WFS1 as a diabetes-related gene and then conducted an extensive investigation focusing on WFS1 in the context of EC. First, we identified WFS1 as the target gene and obtained EC data from the TCGA database. Then, comprehensive analyses and verification experiments, including differential expression analysis, prognostic modeling, functional enrichment analysis, gene mutation profiling, assessment of immune cell infiltration, immunophenoscore (IPS), tumor stemness index scoring, drug sensitivity analysis, single-cell transcriptomic analysis, glycolytic pathway analysis, and clinical verification, were performed to comprehensively evaluate the clinical value of WFS1 in EC.

**Results:**

The EC group had significantly lower WFS1 expression, with an AUC of 0.857 for the ROC diagnostic curve. Overall survival analysis revealed that WFS1 was an independent risk factor for EC; low WFS1 expression was correlated with a poor prognosis. Stemness index analysis revealed that decreased WFS1 expression was associated with increased tumor grade and enhanced tumor stemness, suggesting increased malignancy of EC. In addition, WFS1 expression was correlated with tumor microenvironment features such as immune cell infiltration. WFS1 was also associated with tumor drug resistance.

**Conclusion:**

EC patients with low WFS1 expression have a worse prognosis. WFS1 can be used as diagnostic and prognostic marker for EC.

## 1 Introduction

There have been substantial increases in both the incidence and mortality rates of endometrial cancer (EC), a gynecological malignancy, in recent years, making it one of the most prevalent gynecological malignancies in developed countries (1). Traditionally, EC has been categorized into two primary types on the basis of pathological criteria, namely, hormone-dependent type I EC and hormone-independent type II EC (2). However, recent scientific advancements have prompted the World Health Organization to recommend a shift from histological classification to molecular subgrouping. Notably, a novel classification system encompassing POLEmut, MMRd, NSMP, and p53abn has been proposed, offering valuable insights for tailored EC treatment and improved prognosis prediction (3–5). Furthermore, guidelines such as ESGO/ESTRO/ESP integrate molecular typing, emphasizing the potential efficacy of molecular detection methods in guiding the diagnosis, treatment, and prognosis of EC (6).

Diabetes mellitus (DM) encompasses a heterogeneous group of endocrine disorders primarily characterized by disrupted glucose metabolism, hyperglycemia, insulin resistance, and exacerbated insulin deficiency (7). Epidemiological evidence reveals that it is a significant contributor to global mortality and ranks ninth among the leading causes of death worldwide (8, 9). Furthermore, accumulating evidence suggests that compared with that of the general population, the risk of developing or dying from cancer is markedly higher in diabetic patients (10, 11). Along with the high and increasing incidence of diabetes, which affects one in eleven individuals, there has been a concurrent increase in the number of malignant tumor cases. Patients with concurrent diabetes and malignancy often have a poorer prognosis than those without diabetes, and this phenomenon is particularly prevalent in those with EC (12–16). Diabetic patients with have hazard ratios of 1.65 (95% CI: 1.50–1.81) and 1.32 (95% CI: 1.13–1.55) for EC development and EC-related mortality, indicating significantly increased risk in these patients (16).

In light of the importance of diabetes in the context of EC, it is imperative to delve deeper into the intricate relationship between diabetes and EC risk, particularly within the purview of contemporary molecular-stage disease research. The elucidation of their associations at the molecular level is of paramount importance. Therefore, by utilizing the Comparative Toxicogenomics Database (CTD), GeneCards, and Gene Set Enrichment Analysis (GSEA) databases, we successfully identified the WFS1 gene, a crucial player in both EC and diabetes. By leveraging the TCGA database, we conducted a comprehensive analysis of the prognostic and immune implications of the diabetes-associated gene WFS1 in EC. Notably, our findings revealed significant differences in WFS1 expression between EC tissue and adjacent or normal endometrial tissue, suggesting the potential of the WFS1 gene as a biomarker for EC diagnosis and prognosis evaluation. By integrating data from the TCGA and Gene Expression Omnibus (GEO) and utilizing advanced bioinformatics methods, we performed a comprehensive analysis of the relationship between WFS1 gene expression and EC, considering differential expression, prognostic modeling, functional enrichment analysis, gene mutation profiling, assessment of immune cell infiltration, IPS, tumor stemness index scoring, drug sensitivity analysis, single-cell transcriptomic analysis, glycolytic pathway analysis, and clinical validation. This study offers valuable insights into the molecular-level diagnosis, treatment, and prognosis evaluation of patients presenting with concurrent diabetes and EC, paving the way for more targeted and effective therapeutic strategies.

## 2 Materials and methods

### 2.1 Identification of diabetes-related genes and download of sample data from the TCGA and GEO databases

Using the CTD database (https://ctdbase.org/), we initially retrieved a comprehensive dataset containing information on 281,907 results. Through the process of eliminating redundant data duplicates, we subsequently narrowed this pool to 38,935 genes. Among these genes, we meticulously selected the top 200 genes according to the “Inference Score” parameter. Concurrently, we accessed the GeneCards database (https://www.genecards.org/) to obtain 15,803 diabetes-related genes and selected the top 200 genes on the basis of the “Score” parameter. Additionally, we accessed the “HP_TYPE_II_DIABETES_MELLITUS” dataset from the GSEA database (https://www.gsea-msigdb.org/gsea/index.jsp) and conducted GSEA to identify 192 diabetes-related genes. By intersecting the above gene sets retrieved from three prominent databases, namely CTD, GeneCard, and GSEA, a comprehensive compilation of 22 genes was identified as being intricately associated with diabetes (**Supplementary Table 1**). Subsequent differential and survival analyses were conducted on the entirety of these 22 diabetes-associated genes. Notably, WFS1 emerged not only as a pertinent entity associated with diabetes in the context of EC but also as the most biologically significant candidate among the screened genes. The findings of these analyses highlighted the noteworthy role of WFS1 in the pathogenesis of EC, thus warranting its deliberate selection for further investigation (**Supplementary Image**). Furthermore, to gain insights into the transcription of WFS1 and its association with cancer, we obtained WFS1 transcription expression data along with corresponding clinical information from the official TCGA website. We downloaded RNA-Seq gene expression data generated via the gene expression quantification method and subsequently converted these data into TPM format, facilitating subsequent analysis of WFS1 gene expression in cancer. To ensure the reliability of the expression differences, we validated the expression of the WFS1 gene in EC via the GSE17025, GSE63678, and GSE115810 datasets from the GEO database (https://www.ncbi.nlm.nih.gov/geo/).

### 2.2 Sequencing data for EC

We obtained a total of 589 RNA-Seq data files comprising 554 EC samples and 35 adjacent noncancer samples from the TCGA database (https://portal.gdc.cancer.gov/). Following the removal of duplicate files, the dataset consisted of RNA-seq data files for 545 EC samples and 23 adjacent noncancer samples. The pertinent clinical information, including age, grade, stage, BMI, etc., was preserved for subsequent analyses. Overall survival (OS) data for EC patients were extracted from a study by Liu et al. published in Cell (17). Additionally, mutation data for 519 patients were retrieved from the TCGA database to conduct mutation analysis and calculate the tumor mutation burden (TMB).

### 2.3 Construction of survival models

By employing a comprehensive set of multiple Cox predictions that incorporate variables such as age, grade, and WFS1 gene expression, we utilized regression modeling strategies (RMSs) to construct nomogram plots for 1-year, 3-year, and 5-year survival as well as other pertinent clinical data. By assigning scores to multiple factors, we established a predictive model for EC patient survival on the basis of these composite scores.

### 2.4 Identification and GO analysis, KEGG analysis, and GSEA of WFS1-related differentially expressed genes

The EC samples were segmented into high-level and low-level groups on the basis of the median expression level of WFS1. We subsequently conducted a comprehensive analysis, including differential expression analysis, as well as Gene Ontology (GO) analysis, Kyoto Encyclopedia of Genes and Genomes (KEGG) analysis, and GSEA, to elucidate the functional implications of the differentially expressed genes.

### 2.5 Construction of the PPI network and gene set enrichment analysis

This study utilized the STRING network data platform (version 12) for the identification of protein associations and construction of protein-protein interaction networks. With a specified required score of 0.400, an analysis of WFS1-related genes was conducted, culminating in the development of a protein-protein interaction network (18). Furthermore, these genes were subjected to gene enrichment analysis to evaluate their involvement in specific functions through GO and KEGG enrichment analyses.

### 2.6 Single-cell transcriptomic analysis

To investigate the cellular context of WFS1 as a prognostic marker gene for EC, single-cell transcriptomic analysis was performed via TISCH (http://tisch.comp-genomics.org), and the UCEC-GSE 139555 and UCEC-GSE 154763 single-cell datasets were selected to study WFS1 expression in a variety of immune cells. WFS1 expression and its effect on the tumor microenvironment were clarified.

### 2.7 Gene mutation and stemness index analysis

We downloaded EC gene mutation data from the TCGA database, utilized maftools and dplyr for the extraction and analysis of WFS1-related mutation data. Furthermore, via the TIMER database (https://cistrome.shinyapps.io/timer/), we investigated immune cell infiltration patterns in EC under various copy number alteration (CNA) scenarios associated with the presence of WFS1. The stemness index, which reflects the cellular regeneration and repair capabilities typically observed in stem cells, was calculated via the logistic regression machine learning algorithm OCLR by Malta et al. to evaluate the malignancy of tumor cells (19). A higher stemness index is closely linked to tumor dedifferentiation (20). Therefore, we applied Hao Lian et al.’s approach to represent the stemness index, incorporating the same Spearman correlation (RNA expression data), followed by linear transformation to normalize the stemness index within the [0, 1] range (21).

### 2.8 Relationship between WFS1 gene expression and immune checkpoints in EC

Immune checkpoints are crucial regulatory molecules within the immune system, mainly expressed on immune cells, and they play a significant role in modulating the timing and intensity of immune responses. Therefore, we selected twelve genes associated with immune checkpoints, including SIGLEC15, TIGIT, CD274, HAVCR2, PDCD1, CTLA4, LAG3, PDCD1LG2, FGL1, LGALS9, CEACAM1, and HMGB1. We extracted the expression values of these genes to investigate the relationship between their expression levels and the expression of WFS1 (22–25). The expression data for these genes were obtained from **Supplementary Table 2**. Furthermore, we utilized TIMER 2.0 (http://timer.cistrome.org/) to analyze the correlation between the expression of these genes and that of WFS1, thus elucidating the relationships between the WFS1 gene and immune checkpoints. Ultimately, we used the “circlize” package along with the “hg18” human chromosome data to construct a comprehensive chromosomal localization map illustrating the distribution of WFS1 and immune checkpoint-related genes across the chromosomes.

### 2.9 Analysis of WFS1 gene expression and tumor-infiltrating lymphocyte levels

Immune cells play a critical role in facilitating tumor immune escape and conferring tumor resistance. In this study, we used the TISIDB platform (http://cis.hku.hk/TISIDB/) to analyze the relationship between the expression of the WFS1 gene and TILs by employing a Spearman test for statistical assessment.

### 2.10 WFS1 gene and IPS

Immune checkpoint-targeting drugs significantly influence immune checkpoint mechanisms within tumors. In our investigation, we obtained IPS data for EC from the TCIA database (https://tcia.at/home) and analyzed the relationships between the expression of WFS1 and that of PD1 and Ctla4. This analysis was instrumental in assessing the efficacy of immune checkpoint inhibitor therapies.

### 2.11 Analysis of WFS1 gene and drug sensitivity results

To further investigate the pharmacological utility of the WFS1 gene, we used the CellMiner database (https://discover.nci.nih.gov/cellminer/CellMiner) to download the NCI-60 drug sensitivity and gene expression data. Through the analysis of the correlation between WFS1 gene expression and sensitivity to anti-tumor drugs, we elucidated the relationship between different levels of WFS1 expression and sensitivity to commonly used anti-tumor agents. This insight may aid clinicians in making informed decisions when selecting appropriate anti-tumor therapies.

### 2.12 Clinical validation of the WFS1 gene in pathological tissues of EC patients

To verify the expression of WFS1 in patients, we collected tumor tissue (44 cases) and paracancerous tissue (34 cases) from patients diagnosed with EC via postoperative pathology. Semiquantitative analysis of WFS1 expression was performed via immunohistochemistry (IHC). IHC procedures were performed in strict accordance with the instructions of the WFS1 antibody (IHCeasy WFS1 Ready-To-Use IHC Kit, ProteinTech, Cat No: KHC0048). WFS1 expression was evaluated by a professional pathologist as well as ImageJ software (version 1.54, IHC Profiler) and scored via a scoring system (26, 27). The intensity of staining (no staining=0, weak staining=1, moderate staining=2, strong staining=3) and the percentage of stained cells (0–5%=0, 5–25%=1,26–50%=2, 51–75%=3, 76–100%=4) were scored, and these scores were multiplied to obtain the final score (28).

### 2.13 Statistical analysis

Statistical analyses were conducted via R software (version 4.2.1). The KM survival analysis results across different groups were derived through Cox regression utilizing the “survival” and “survminer” packages. The GSE17025, GSE63678, and GSE115810 datasets, consisting of 122 EC samples and 20 normal samples, was utilized to validate WFS1 expression. To compare differences among three or more groups, the Kruskal-Wallis test (Multiple Comparisons: dunn’s test) was used (29). Box plots were generated via the “ggplot2” package. Differences between two groups were determined via Wilcoxon. (P<0.05*, P<0.01**, P<0.001***).

## 3 Results

### 3.1 Pan-cancer analysis of WFS1 expression

**Figure 1:**
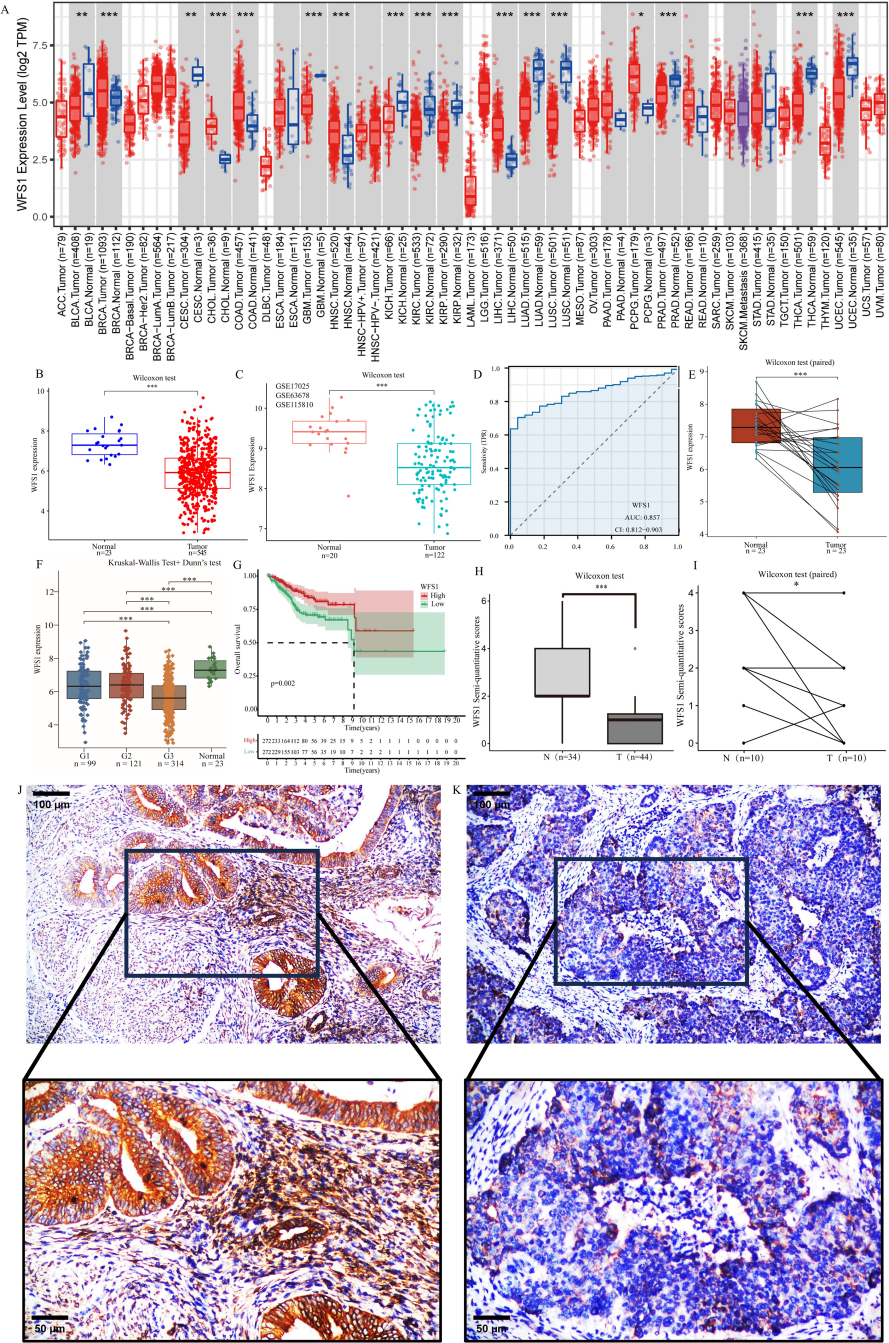
Differential expression of WFS1 in tumors and survival analysis. **(A)** Expression of WFS1 in pan-cancer. (*p < 0.05, **p < 0.01, and ***p < 0.001). **(B)** The mRNA expression levels of WFS1 in EC and normal. **(C)** The mRNA expression levels of WFS1 in EC and normal groups (GSE17025, GSE63678 and GSE115810 datasets). **(D)** Expression levels of WFS1 are used to assist in the diagnosis of EC. **(E)** The mRNA expression levels of WFS1 in EC and matched-adjacent normal. **(F)** The mRNA expression levels of WFS1 in different grades EC and normal. **(G)** KM curves showed shorter OS in the WFS1 low expression group. **(H, I)** The EC group and the paracancerous tissues were subjected to comparative analysis through IHC scoring. **(H)** indicates an overall difference. **(I)** indicates compared differences. **(J)** IHC of WFS1 in normal tissues (upper: 100um, lower: 50um). **(K)** IHC of WFS1 in tumor tissues (upper: 100um; lower: 50um).

**Figure 1A** shows the expression pattern of the WFS1 gene across diverse tumor types. The white area in the figure exclusively represents gene expression in the tumor group, whereas the gray area represents expression in both the tumor and normal groups. Our findings revealed notable differences in WFS1 gene expression among 17 types of tumors, with a decrease in expression observed in 11 types and an increase in 6 types.

### 3.2 Expression of WFS1 in EC and normal tissues

To further evaluate the diagnostic value of WFS1 in EC, we assessed 545 EC samples and 23 control samples and observed a significant decrease in WFS1 expression in the EC group (**Figure 1B**). This finding is consistent with the results obtained from the analysis of the GSE17025, GSE63678 and GSE115810 datasets (**Figure 1C**), where WFS1 expression was also found to be lower in the EC group. Additionally, paired analyses were conducted on tumor tissues and adjacent nontumor tissues from the same patient (**Figure 1E**), which revealed significant differences in WFS1 expression. Notably, WFS1 exhibited substantial differential expression between the EC group and the control group, characterized by low WFS1 expression in cancer tissues. We categorized patients on the basis of tumor grade and analyzed WFS1 expression across different tumor grades (**Figure 1F**). The results suggested that, except for the G1 and G2 stages, significant differences were present in all other groups, with higher tumor grades being associated with lower WFS1 gene expression and increased risk levels. In order to further verify the outcomes of the aforementioned database analysis, samples of EC tissues and paracancerous tissues were selected for examination, with the findings subsequently confirmed through IHC validation. As anticipated, WFS1 exhibited elevated expression levels in paracancerous tissues (**Figure 1J**), while demonstrating lower expression in EC tissues (**Figure 1K**). Subsequent semi-quantitative analysis further elucidated a statistically significant discrepancy in expression levels between the two tissue groups (P<0.05; **Figures 1H, I**). In summary, the research indicates that WFS1 is lowly expressed in EC when compared to normal tissues, and individuals with relatively lower WFS1 expression levels are more prone to poorly differentiated tumors. Therefore, the assessment of WFS1 levels may serve as a valuable tool for clinicians in both initial risk evaluation and definitive diagnosis of EC.

### 3.3 Significance of WFS1 as a diagnostic and prognostic marker for EC

To further explore the diagnostic and prognostic value of WFS1 levels in patients with EC, based on the WFS1 expression profiles of both the EC and control groups, a diagnostic ROC curve was constructed (**Figure 1D**). This ROC curve revealed sensitivity and specificity values for WFS1 of 0.7046 and 0.9565, respectively, and demonstrated an AUC value of 0.857 (0.812–0.903). Furthermore, the patients in the EC group were stratified into subgroups on the basis of the median WFS1 expression level, and subsequent Kaplan-Meier (KM) curve analysis was subsequently performed (**Figure 1G**). The results revealed significant correlation (HR=0.52, P=0.002) between high WFS1 expression and a favorable prognosis in terms of OS. This finding provides additional evidence supporting the utility of WFS1 as a diagnostic marker for EC, and underscores its potential prognostic value. Specifically, individuals with EC exhibiting low levels of WFS1 expression may be associated with a poorer prognosis.

### 3.4 Correlations between WFS1 levels and clinical features of EC and establishment of a prognostic model

**Figure 2:**
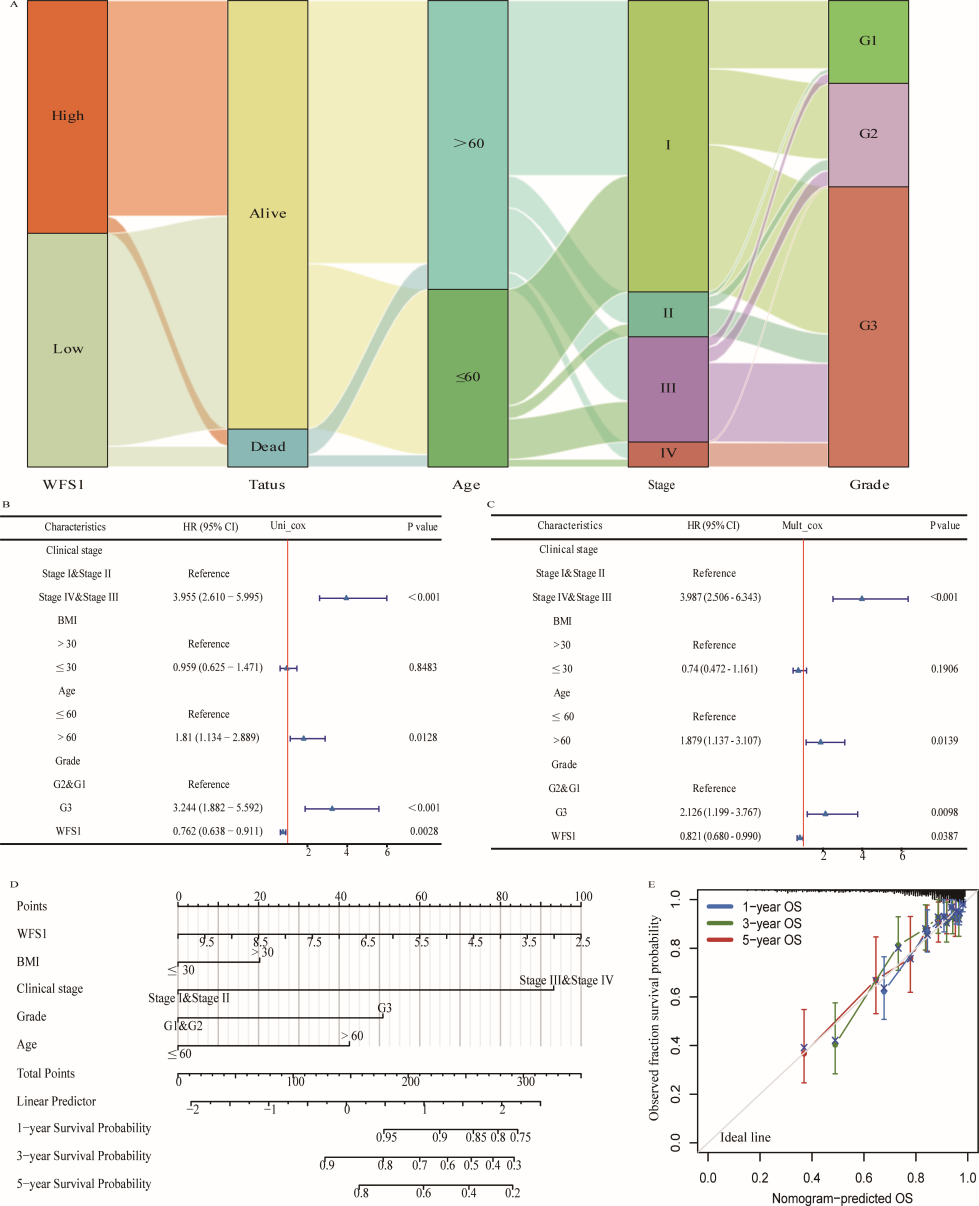
Prognostic modeling and statistical analysis. **(A)** Sankey diagram based on clinical data. Cox regression analysis of WFS1expression and related clinical features in overall survival of EC patients. **(B)** Univariate Cox regression analysis. **(C)** Multivariate Cox regression analysis. **(D)** WFS1 and clinical information for predicting prognosis in EC patients. **(E)** Calibration curves of the nomogram predicting OS in patients with EC.

To investigate the correlation between WFS1 gene expression and the clinical features of patients with EC, we stratified data from 545 patients with EC into two cohorts on the basis of the median WFS1 gene expression level. The cohort with low WFS1 gene expression comprised 272 patients, whereas the cohort with high WFS1 gene expression comprised 273 patients. We conducted a chi-square test to compare the two cohorts. The results, as shown in **Supplementary Table 3**, revealed significant differences in terms of mortality (P=0.002), tumor grade (P<0.0001), age (P=0.005), and stage (P=0.030). These findings highlight the strong correlations between WFS1 gene expression and various clinical features, indicating its potential as a prognostic factor and risk indicator for patients with EC. We further utilized these data to construct a Sankey diagram, offering a comprehensive visual representation of these clinical characteristics (**Figure 2A**). Additionally, we performed single-factor analysis (**Figure 2B**) and multifactor analysis (**Figure 2C**) considering factors such as age, grade, TNM status, WFS1, BMI, etc., which enabled the development of a nomogram (**Figure 2D**). As shown in **Figure 2D**, each variable was assigned a score ranging from 0 to 100, and the total score represented the cumulative score of all the variables. By mapping the total score onto the corresponding survival curve, we can predict the probability of patient survival over time. The high contribution of WFS1 expression to the risk score underscores its pronounced influence on the prognosis of EC. Furthermore, to validate the model’s accuracy, we established models for 1-, 3-, and 5-year prognosis prediction (**Figure 2E**). Collectively, the prognostic model integrating WFS1 expression levels and pertinent clinical patient data facilitates prognostication of survival outcomes among patients with EC, thus offering enhanced guidance to healthcare practitioners in treatment decision-making and prognosis assessment.

### 3.5 Differential analysis and functional enrichment of WFS1

**Figure 3:**
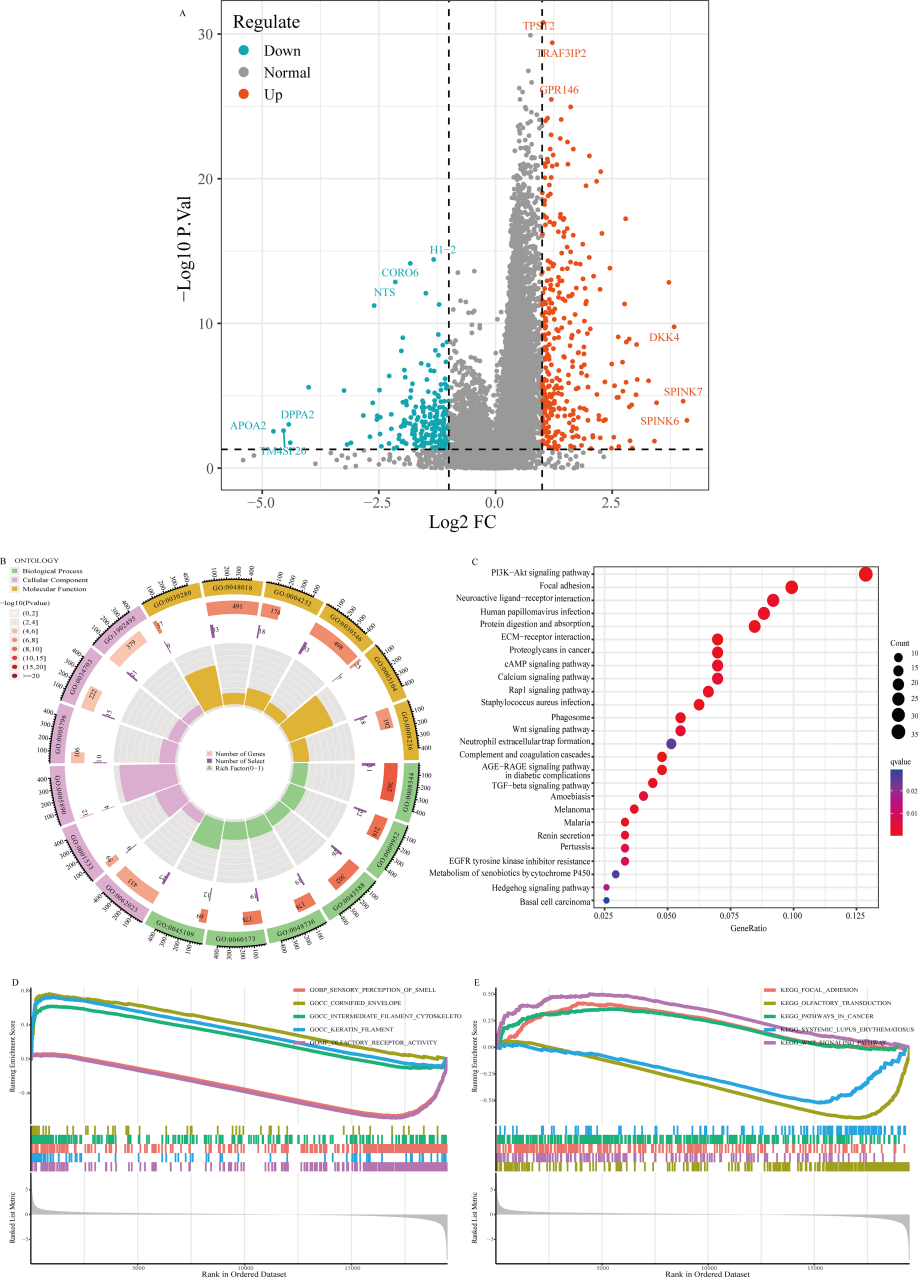
Volcano plot of gene expression and enrichment analysis. **(A)** Volcano plot visualization of gene expression differentials based on WFS1 expression. **(B)** GO enrichment analysis. **(C)** KEGG enrichment analysis. **(D, E)** GESA-based enrichment analysis: GO enrichment **(D)** and KEGG enrichment **(E)**.

We categorized the 545 EC patients into two groups on the basis of the median expression level of WFS1. Following this categorization, we performed a comparative analysis of gene expression profiles and generated a volcano plot to visualize the results (**Figure 3A**). To investigate the functional implications of these differentially expressed genes in EC across varying levels of WFS1 expression, thereby elucidating the biological functions associated with the differential gene expression in the high and low WFS1 expression groups, the differentially expressed genes were then subjected to GO enrichment analysis (**Figure 3B**) and KEGG enrichment analysis (**Figure 3C**). The top six enriched biological process (BP) terms included epidermis development, anterior/posterior pattern specification, skin development, appendage development, limb development, and intermediate filament organization. The top six enriched cellular component (CC) terms were collagen-containing extracellular matrix, cornified envelope, sodium-potassium-exchanging ATPase complex, Golgi lumen, cation channel complex, and transmembrane transporter complex. Furthermore, the top six enriched molecular function (MF) terms were structural constituent of the skin epidermis, receptor-ligand activity, serine-type endopeptidase activity, signaling receptor activator activity, fibroblast growth factor receptor binding, and serine-type peptidase activity. In terms of KEGG pathways, the top five pathways were the PI3K-Akt signaling pathway, focal adhesion, neuroactive ligand-receptor interaction, human papillomavirus infection, protein digestion, and absorption. GSEA revealed that the top five GO terms were sensory perception of smell, cornified envelope, intermediate filament cytoskeleton, keratin filament, and olfactory receptor activity (**Figure 3D**). The top five KEGG pathways identified were focal adhesion, olfactory transduction, pathways in cancer, systemic lupus erythematosus, and the Wnt signaling pathway (**Figure 3E**). The results suggest that signaling pathways such as PI3K-Akt may play a role in the poorer prognosis of the low WFS1 expression group in EC. In summary, a plethora of genes exhibit differential expression between the WFS1 low-expression cohort and the high-expression cohort. The multiple biological functions and interactions among the genes associated with WFS1 may potentially contributing to the unfavorable prognosis observed in the cohort with low WFS1 expression in EC, thus shedding light on prospective avenues for further research.

### 3.6 PPI network and gene enrichment analysis

**Figure 4:**
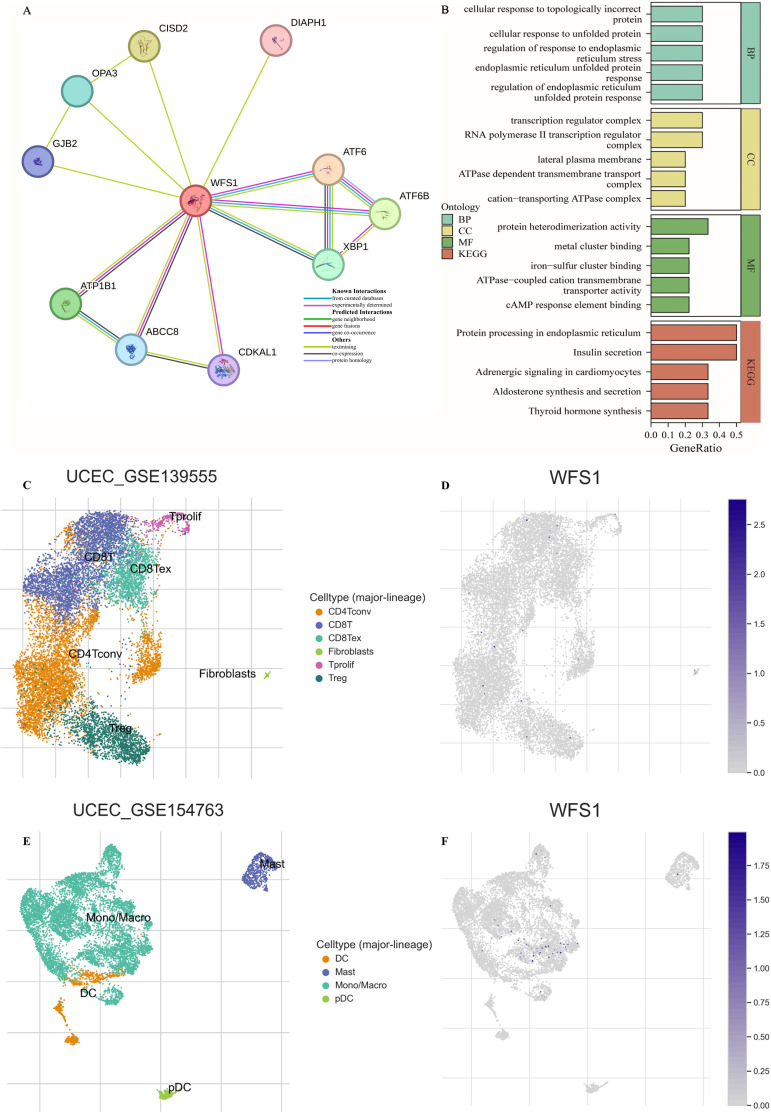
PPI network, feature enrichment analysis and single-cell transcriptomic analysis. **(A)** Network of interacting genes involving WFS1. The circles represent query proteins and their corresponding first shell interactors in the network. The color and number of edges represent the source and amount of supporting evidence respectively. **(B)** Functional enrichment analysis of interacting genes. **(C, E)** Distribution of UCEC-GSE 139555 and UCEC-GSE 154763 immune cells. **(D, F)** Expression of WFS1 in various immune cells.

Protein interaction networks serve as crucial tools in elucidating the functional roles of genes and the underlying biological processes they participate in. Using the STRING database, we constructed a protein interaction network centered on WFS1 (**Figure 4A**). The identified genes, including DIAPH1, ATF6, ATF6B, XBP1, CDKAL1, ABCC8, ATP1B1, GJB2, OPA3, and CISD2, were subjected to GO and KEGG enrichment analyses, as depicted in **Figure 4B**. The enriched BPs included the regulation of the endoplasmic reticulum unfolded protein response, the endoplasmic reticulum unfolded protein response, the regulation of the response to endoplasmic reticulum stress, the cellular response to unfolded protein, and the cellular response to topologically incorrect protein. The top enriched CCs were the RNA polymerase II transcription regulator complex, transcription regulator complex, cation-transporting ATPase complex, ATPase-dependent transmembrane transport complex, and lateral plasma membrane. Moreover, the top five enriched MFs of these genes were protein heterodimerization activity, cAMP response element binding, ATPase-coupled cation transmembrane transporter activity, iron-sulfur cluster binding, and metal cluster binding. The top five significantly enriched KEGG pathways were insulin secretion, protein processing in the endoplasmic reticulum, thyroid hormone synthesis, aldosterone synthesis and secretion, and adrenergic signaling in cardiomyocytes. Based on the above findings, it becomes evident that WFS1 plays a pivotal role in a variety of biological processes, thereby offering valuable insights to steer and catalyze future investigations on the mechanistic actions of WFS1 in EC.

### 3.7 Single-cell transcriptomic analysis

To further analyze the effect of WFS1 on the tumor microenvironment, single-cell transcriptome analysis was performed. As shown in **Figures 4C-F**, the WFS1 gene was expressed only in small amounts in monocytes, which are important immunomodulatory cells in the human body that influence tumor escape as well as tumor immunity. In addition, the WFS1 gene was hardly expressed in other immune cells in EC tumor tissues, which may be one of the reasons for the worse prognosis of EC patients in the WFS1 low-expression group.

### 3.8 Gene mutation, CNA analysis and the tumor stemness index

**Figure 5:**
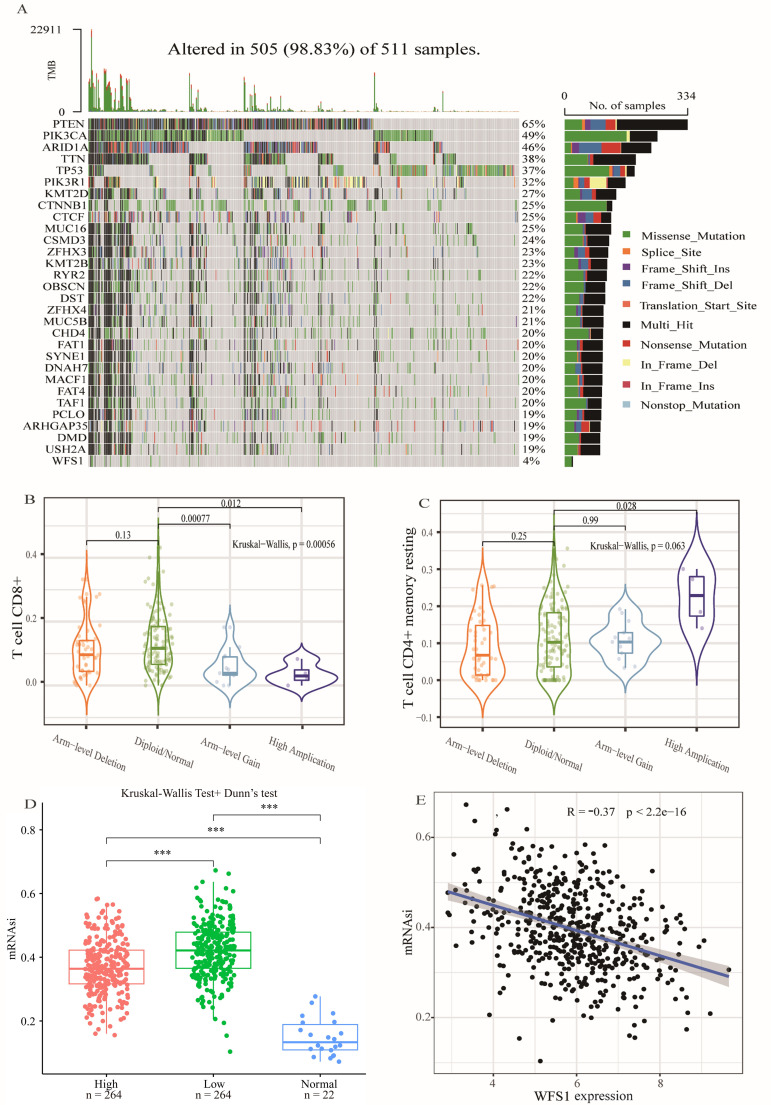
Gene mutation, CNA analysis and the tumor stemness index. **(A)** Gene mutation analysis in EC. **(B, C)** Effects of various copy number alteration (CNA) conditions on T cell CD4+ memory quiescence **(B)** and T cell CD8+ **(C)**. **(D)** Analysis of tumor stemness index based on WFS1 expression grouping. Effect of different WFS1 expression levels on tumor stemness. **(E)** Linear relationship between WFS1 expression level and tumor stemness. (***p < 0.001).

Gene mutations are known to play pivotal roles in the development of tumors. In this study, utilizing data from the TCGA database, we obtained EC mutation data and conducted an analysis of gene mutation statuses within EC. A waterfall plot was subsequently generated to visualize the findings (**Figure 5A**). Our analysis revealed that the most frequently mutated gene in EC is PTEN. Specifically, the mutation frequency of WFS1 in EC was 4%, with missense mutations being the predominant type. Furthermore, we investigated the impact of WFS1 under various copy number alteration (CNA) types, including arm-level deletion, diploid/normal, arm-level gain, and high amplification, on T-cell CD4+ memory resting (**Figure 5B**) and T-cell CD8+ populations (**Figure 5C**). Notably, significant differences were observed in CD4+ memory and CD8+ T cells between the high amplification and diploid/normal CNA conditions, suggesting that some WFS1 CNAs may influence immune cells. Moreover, we assessed the influence of WFS1 on tumor stemness by conducting tumor stemness index analysis on the basis of WFS1 expression grouping (**Figure 5D**). Our analysis revealed marked differences (P<0.001) between the high-expression and low-expression groups, as well as between both the EC group and the normal group (P<0.001). Among the three groups, the normal group presented the lowest stemness index, whereas the WFS1 low-expression group presented the highest index. These findings are consistent with experimental predictions indicating that the low WFS1 expression group possesses stronger tumor dedifferentiation capabilities. Furthermore, we constructed a linear correlation graph for WFS1 and mRNAsi (**Figure 5E**), revealing a negative correlation between WFS1 gene expression and mRNAsi (ρSpearman = -0.36). Lower WFS1 expression corresponded to a higher mRNAsi score, indicative of a greater degree of malignancy. The research revealed that WFS1 mutations were present in a mere 4% of EC and did not exhibit a substantial impact on the pathogenicity of EC. In addition, the results of tumor stemness analysis further indicated a heightened level of stemness in the group exhibiting decreased WFS1 expression, which was consistent with **Figure 1E**. Furthermore, The degree of tumor differentiation was notably reduced in the subset characterized by low WFS1 expression.

### 3.9 Analysis of immune checkpoints and immune infiltration

**Figure 6:**
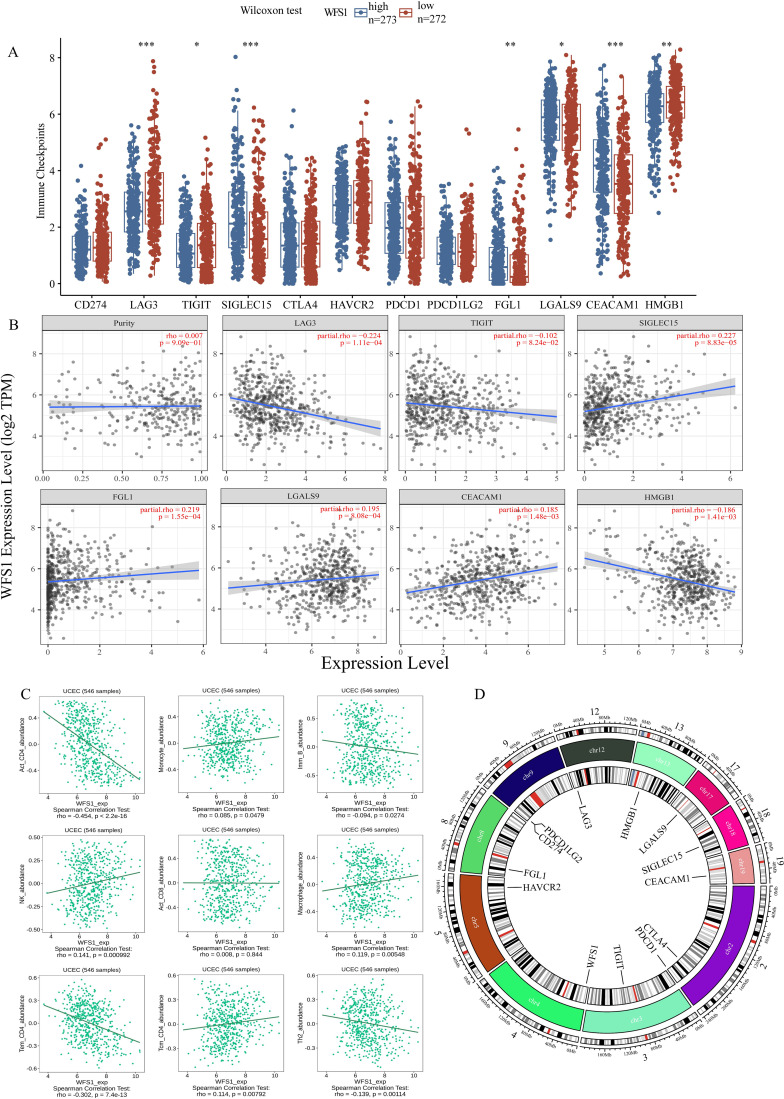
WFS1 expression on tumor immunity. **(A)** Differences in common immunodetection sites across different subgroups of WFS1 expression. **(B)** Effect of WFS1 expression level on the expression of LAG3, TIGIT, SIGLEC15, FGL1, LGALS9, CEACAM1, and HMGB1. **(C)** Effects of WFS1 on common immune cells. **(D)** The chromosomal locations of WFS1 and twelve immune checkpoint-related genes. The outermost circle of the diagram represents a scale of chromosomal length, while the innermost circle indicates the specific positions of the genes on the chromosomes. Different colors within the chromosomes indicate various segment partitions, with the red section specifically denoting the location of the centromere. (*p < 0.05, **p < 0.01, and ***p < 0.001).

Tumor immune escape represents a significant area of investigation within the field of oncological therapy, with the examination of immune checkpoints and immune cell functions serving as critical components in elucidating the intricacies of this phenomenon. To further delve into the specific mechanisms of WFS1 in tumor immune evasion, this study undertook a comparative analysis of the expression levels of prevalent immune checkpoint-related genes across cohorts distinguished by high and low WFS1 expression, aiming to delineate the potential associations between immune checkpoint-related genes and WFS1. Specifically, we conducted a comparative analysis of 12 commonly recognized immune checkpoint-related genes (**Figure 6A**). Among these genes, significant differences in expression levels were observed for LAG3, TIGIT, SIGLEC15, FGL1, LGALS9, CEACAM1, and HMGB1 between the two groups. To further investigate the correlation between WFS1 expression and the aforementioned immune checkpoint-related genes, we used the TIMER database to conduct a Spearman correlation analysis and constructed linear correlation graphs accordingly (**Figure 6B**). Our results revealed a negative correlation between WFS1 expression and LAG3 (ρSpearman = -0.224), TIGIT (ρSpearman = -0.102), and HMGB1 (ρSpearman = -0.186), whereas a positive relationship was observed between WFS1 and SIGLEC15 (ρSpearman = 0.227), FGL1 (ρSpearman = 0.219), LGALS9 (ρSpearman = 0.195), and CEACAM1 (ρSpearman = 0.185). Furthermore, leveraging the TISIDB database, we explored the associations between WFS1 gene expression and various immune cells (**Figure 6C**) by analyzing Act_CD8, Act_CD4, Tcm_CD4 (central memory T cells), Tem_CD4, Imm_B (immature B cells), macrophages, monocytes, and NK cells. Our analysis revealed associations between WFS1 gene expression and Act_CD4 (r=-0.454, P<2.2e-16), Tcm_CD4 (r=0.114, P=0.00792), Tem_CD4 (r=-0.302, P=7.4e-13), Imm_B (r=-0.094, P=0.0274), macrophages (r=0.119, P=0.00548), monocytes (r=0.085, P=0.0479), and NK cells (r=0.141, P=0.000992), with no significant correlation with Act_CD8 (r=0.008, P=0.844). It is noteworthy that WFS1 expression is negatively correlated with Act_CD4 and Tem_CD4, while positively correlated with Tcm_CD4. Act_CD4 can differentiate into both Tem_CD4 and Tcm_CD4 subsets. Given the predominant localization of Tcm_CD4 within secondary lymphoid organs, a decrease in Tcm_CD4 cell attributed to low WFS1 expression may contribute to an increased likelihood of tumor tissue metastasis to the lymph nodes (30). Collectively, these results demonstrate a close association between the WFS1 gene and not only immune checkpoint-related genes including LAG3, TIGIT, SIGLEC15, FGL1, LGALS9, CEACAM1, and HMGB1, but also with the infiltration of immune cells within tumors. These findings underscore the broad involvement of WFS1 with various immune checkpoints and immune cells, showcasing its significant research value in the context of tumor immune evasion. The investigation of gene localization on chromosomes facilitates the subsequent development of targeted pharmacological interventions and advances in gene-targeted therapies. Consequently, we have constructed a comprehensive chromosomal localization map illustrating the distribution of WFS1 and immune checkpoint-related genes across the chromosomes (**Figure 6D**). The relationship between WFS1 and immune checkpoints can establish a theoretical foundation for investigating immune checkpoint drugs, thereby offering enhanced guidance for EC therapy.

### 3.10 Analysis of WFS1 and the immune checkpoints PD1 and Ctla4

**Figure 7:**
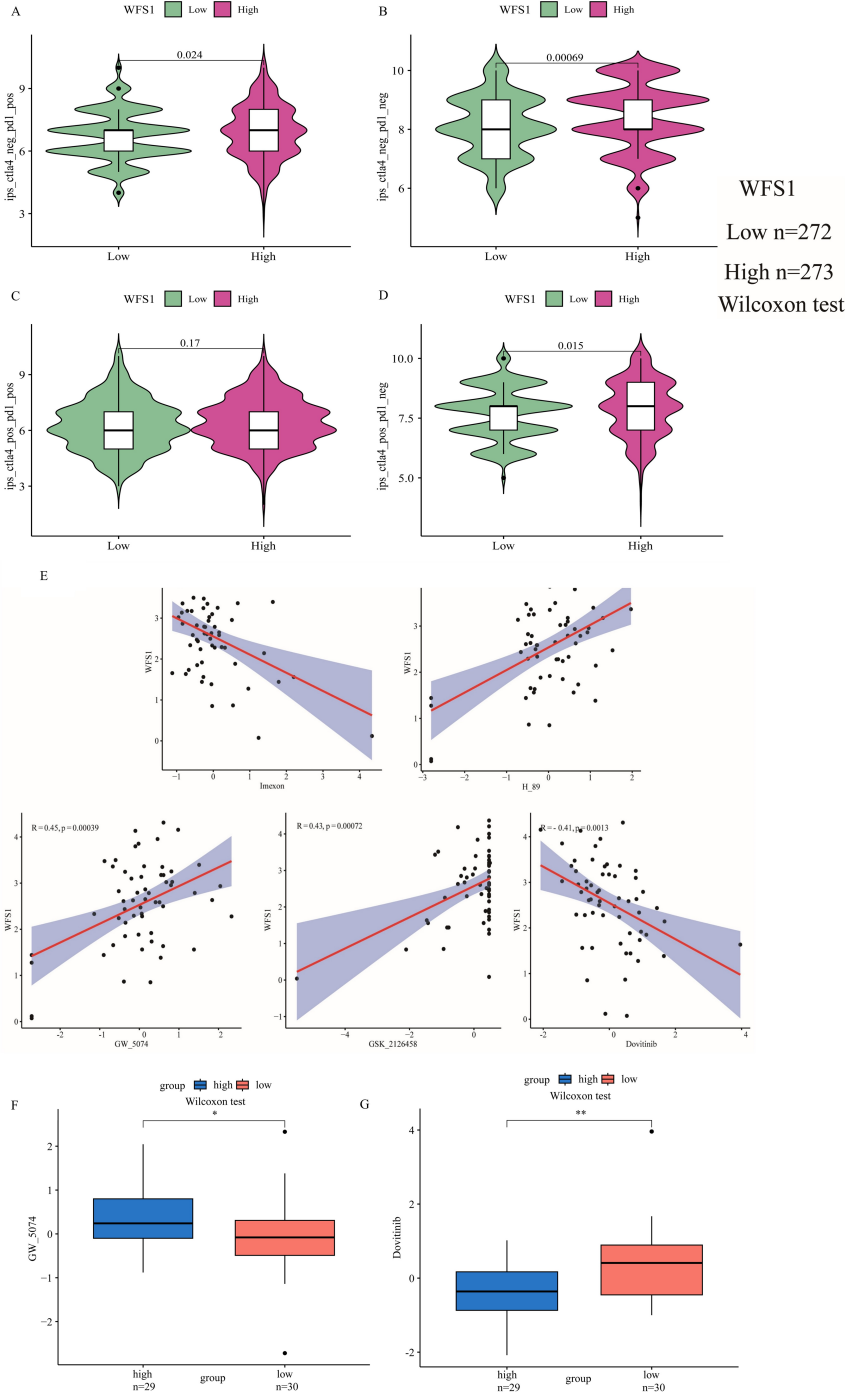
Analysis of the relationship between WFS1 and the immune checkpoints PD1 and Ctla4, as well as WFS1 and tumor drug sensitivity. **(A-D)** IPS scores for four different expression scenarios of PD1 and Ctla4 when categorized into high and low expression groups based on WFS1 expression. **(A)** CTLA-4 negative and PD-1 positive. **(B)** CTLA-4 negative and PD-1 negative. **(C)** CTLA-4 positive and PD-1 positive. **(D)** CTLA-4 positive and PD-1 negative. **(E)** Correlation analysis between WFS1 expression and five drugs (dovitinib, GSK_2126458, GW_5074, H89, and imexon) from left to right. **(F, G)** Drug sensitivity to antitumor drugs in different subgroups of WFS1. (*p < 0.05, **p < 0.01).

To further explore the impact of WFS1 expression on immunotherapy outcomes, we conducted a rigorous investigation employing IPS to assess the efficacy of immune checkpoint inhibitors. Specifically, we focused on two common immune checkpoints: PD1 and Ctla4. Our findings revealed that only when both CTLA-4 and PD-1 were positive (**Figure 7C**) did the expression of WFS1 did not affect treatment sensitivity. However, statistically significant differences in sensitivity were observed in other scenarios, namely, CTLA-4 negative and PD-1 positive (**Figure 7A**), CTLA-4 negative and PD-1 negative (**Figure 7B**), and CTLA-4 positive and PD-1 negative (**Figure 7D**). Notably, the high WFS1 expression group exhibited increased sensitivity to PD1 and Ctla4 immune checkpoint inhibitors. Consequently, these immune checkpoint inhibitors are anticipated to yield superior therapeutic effects in patients with high WFS1 expression levels.

### 3.11 Drug sensitivity

To explore the pharmacological implications of WFS1, we used CellMiner to assess the responsiveness of samples with different expression levels of the WFS1 gene to prevalent anticancer medications and constructed linear correlation graphs (**Figure 7E**). Our analysis included five commonly used anticancer drugs: dovitinib (R = -0.41, P= 0.0013), GSK_2126458 (R = 0.43, P= 0.00072), GW_5074 (R = 0.45, P= 0.00039), H89 (R = 0.52, P= 2.5e−05), and imexon (R = -0.43, P= 0.00072). Furthermore, by stratifying patients on the basis of WFS1 gene expression, we discerned variations in drug sensitivity between the two cohorts, thereby elucidating the impact of WFS1 gene expression on anticancer drug responsiveness. Notably, disparities in sensitivity were observed for dovitinib (P<0.01; **Figure 7G**) and GW_5074 (P<0.05; **Figure 7F**) between WFS1 expression groups. Based on existing research with pharmaceutical agents, these findings further illuminate potential avenues for the advancement of pharmacological approaches by targeting the modulation of WFS1 in EC.

### 3.12 Effect of WFS1 on glycolysis in endometrial cancer

**Figure 8:**
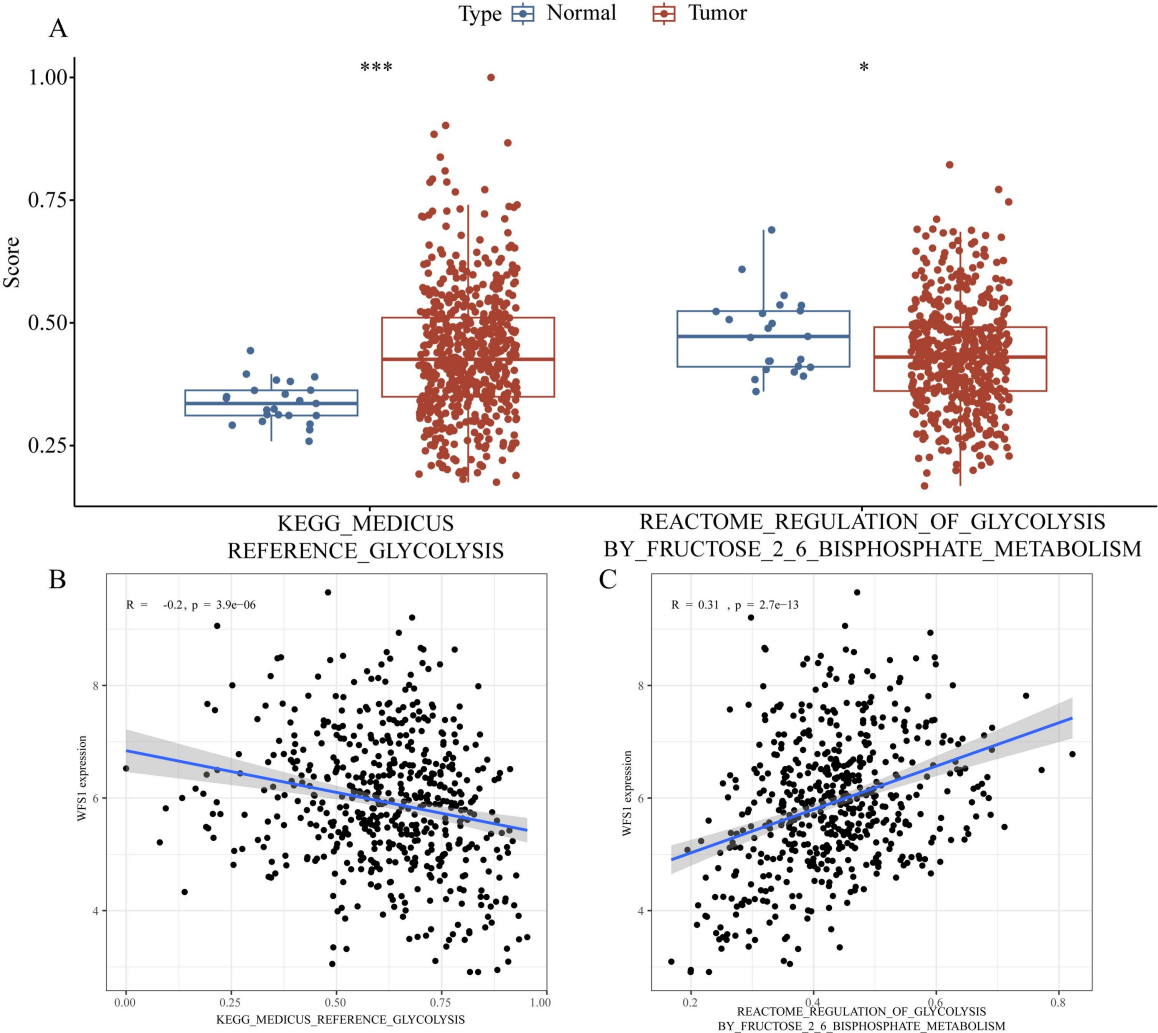
Effect of WFS1 on glycolysis in endometrial cancer. (**A** Left) Comparison of glycolysis scores between tumor and normal groups. (**A** Right) Analysis of differences in the regulation of glycolysis by fructose 2-6 bisphosphate metabolism scores between tumor and normal groups. **(B)** Impact of WFS1 expression on glycolytic scoring. **(C)** Effect of WFS1 expression on the regulation of glycolysis by fructose 2-6 phosphate. (*p < 0.05, ***p < 0.001).

The acquisition of nutrients and the associated metabolic pathways in tumor cells is a dysfunctional process that supports their abnormal growth and metastasis, constituting a fundamental metabolic alteration in cancer (31). One classic aspect of tumor metabolism is the process of glycolysis (32). In diabetic patients, tumor cells exhibit particularly severe rapid growth and metastasis, which can be attributed to the hyperglycemic environment present in these patients. According to the Otto Warburg effect, tumor cells can utilize large amounts of glucose for anaerobic glycolysis to produce sufficient energy, which may explain the accelerated growth and metastasis of EC in diabetic patients. In our study, we found that the expression of the diabetes-associated gene WFS1 was downregulated in EC, affecting various components of the tumor microenvironment and leading to a poorer prognosis for patients. As a gene associated with diabetes, WFS1 plays a pivotal role in modulating the glycolytic pathway in EC. Therefore, we downloaded glycolysis datasets from the GESA database and performed glycolytic pathway scoring on TCGA samples using GSVA, revealing significant differences in the glycolytic metabolic processes between EC and normal endometrial tissue (**Figure 8A**). Further analysis indicated that WFS1 significantly affected the glycolytic processes in EC (**Figure 8B**), with decreased WFS1 expression correlating with higher glycolytic scores (R=-0.2, p=3.9e-06). Higher glycolytic scores typically indicated stronger tumor proliferation characteristics (33). In addition, our study also revealed that WFS1 affected the regulation of fructose 2,6-bisphosphate metabolism on glycolysis in EC (**Figure 8C**). Collectively, these results underscore the influence of WFS1 expression on glycolytic processes in EC cells, thereby influencing tumor metabolic mechanisms. This may be one of the reasons for the observed alterations in the tumor microenvironment of EC.

## 4 Discussion

EC is a prevalent gynecological malignancy anticipated to account for 65,950 new cancer cases and 12,550 related deaths in the United States in 2022 (34). The main therapeutic strategy currently involves surgery, followed by radiotherapy and chemotherapy, as this approach has demonstrated good efficacy in treating early-stage EC (35). Nevertheless, owing to the nonspecific symptoms frequently exhibited by early-stage patients, the majority are diagnosed at advanced stages, where the conventional treatment regimen yields a poor prognosis. Therefore, there is a pressing need for novel methods to aid in early detection and enhance therapeutic strategies for late-stage EC (36). Our comprehensive literature review revealed that EC classification has progressed to the molecular level and is advocated for in numerous treatment guidelines. Furthermore, gene testing schemes have been utilized in the treatment and prognostic assessment of various cancers, such as acute leukemia, lymphoma, and lung cancer. Advances in molecular technology have provided an enhanced foundation and strategies for diagnosing and treating EC (37–40). Consequently, this research project was initiated to provide additional molecular evidence and research strategies for the diagnosis and treatment of EC.

Our study revealed a significant downregulation of WFS1 mRNA expression in EC, particularly in patients with higher grades of malignancy. Through the construction of a diagnostic ROC curve based on WFS1 gene expression, we demonstrated the potential value of WFS1 as an aid in the diagnosis of EC. Further survival analysis confirmed that individuals with lower levels of WFS1 expression experienced shorter OS periods. To elucidate the mechanism underlying this observation, we conducted a comprehensive tumor stemness index analysis in relation to WFS1 expression, revealing a positive correlation between a higher mRNAsi score and increased tumor dedifferentiation ability, indicating an elevated risk in these patients. Furthermore, utilizing the TIMER database, we identified a strong correlation between WFS1 and immune cell infiltration within tumor tissues, which may contribute to the increased risk associated with decreased WFS1 expression. Our assessment of the role of WFS1 in commonly used anticancer drugs revealed its potential relationships with five standard drugs, thereby providing a theoretical foundation for broader indications of these therapies. Finally, through the validation of clinicopathological specimens, we verified that reduced WFS1 expression indeed serves as a key molecular determinant leading to unfavorable outcomes in patients with EC. Collectively, our findings underscore the pivotal role of the diabetes-related gene WFS1 in EC diagnosis and prognosis evaluation, thus positioning WFS1 as a promising therapeutic target for the treatment of this condition.

WFS1, which is located primarily in the endoplasmic reticulum, encodes a specific amino acid glycoprotein. While early research linked mutations in the WFS1 gene to rare Wolfram syndrome, recent studies have revealed a broader role for WFS1, potentially impacting the prognosis of lung cancer, rectal cancer, and colon cancer through its expression levels (25, 41, 42). These effects on patient outcomes may be attributed to endoplasmic reticulum stress and disturbances in glucose metabolism (43–46). In animal models, WFS1 plays a role in crucial biological processes such as biomembrane transport and secretion, with disruptions leading to endoplasmic reticulum stress responses, including apoptosis (47, 48). Additional studies by Takahiro Yamada have also shown a strong correlation between WFS1 and endoplasmic reticulum stress, influencing cell cycle progression and pancreatic B-cell apoptosis (49). These findings suggest that WFS1 could impact tumor prognosis by modulating endoplasmic reticulum stress and glucose metabolism. However, extensive research into the associated pathways and in-depth mechanistic studies involving WFS1 in tumors are lacking. Consequently, a more comprehensive investigation into these aspects is warranted to elucidate the relationship between WFS1 and tumors, particularly with a focus on its connection with EC.

This study has certain limitations that should be noted. While our findings were validated via various databases, further comprehensive exploration of signaling pathways and the implementation of broader experimental approaches in animals and cells are necessary. Therefore, these future efforts will substantially facilitate the potential application of WFS1 in the treatment of EC.

## 5 Conclusion

In this study, we conducted an extensive investigation focusing on the diabetes-associated gene WFS1 in the context of EC. We initially identified WFS1 as the target gene and obtained EC data from the TCGA database. Comprehensive analyses and verification were performed, including differential expression analysis, prognostic modeling, functional enrichment analysis, gene mutation profiling, assessment of immune cell infiltration, IPS, tumor stemness index scoring, single-cell transcriptomic analysis and drug sensitivity analysis. Finally, we utilized the glycolytic pathway score to analyze the effect of WFS1 on glycolytic process in EC. Collectively, these analyses were conducted to provide a comprehensive assessment of the clinical significance of WFS1 within the context of EC. Overall, these analyses were performed to comprehensively evaluate the clinical value of WFS1 in the context of EC.

Additionally, we integrated WFS1 expression with BMI, stage, grade, age, and other clinical factors to predict the 1-, 3-, and 5-year survival rates of patients with EC. Notably, our findings revealed a significant negative correlation between WFS1 expression and the prognosis of EC. Specifically, reduced WFS1 expression was found to be associated with increased tumor grade and enhanced tumor stemness, indicative of increased malignancy in patients with EC. Furthermore, these effects appear to be linked to the influence of WFS1 on immune cell infiltration and the response to anticancer drugs in the context of EC. Moreover, the decreased expression of WFS1 significantly impacted the glycolytic process in EC by disrupting the regulation of glycolysis through fructose 2,6-bisphosphate metabolism. This disruption may further influence the metabolic pathways in EC and serve as a crucial factor contributing to changes in the tumor microenvironment, potentially facilitating immune evasion. Overall, our comprehensive analysis highlights the potential of WFS1, a diabetes-associated gene, as a promising diagnostic and prognostic biomarker for EC. These findings also suggest the potential of WFS1 as a promising target for novel therapeutic interventions, particularly for individuals with concurrent EC and diabetes.

## Data Availability

The raw data supporting the conclusions of this article will be made available by the authors, without undue reservation.
