# Supplementary figures and images for: The immunological and prognostic significance of the diabetes mellitus-related gene WFS1 in endometrial cancer
Source: Front Immunol. 2024 Oct 16;15:1464421. doi: 10.3389/fimmu.2024.1464421 (PMC11521820; doi:10.3389/fimmu.2024.1464421)

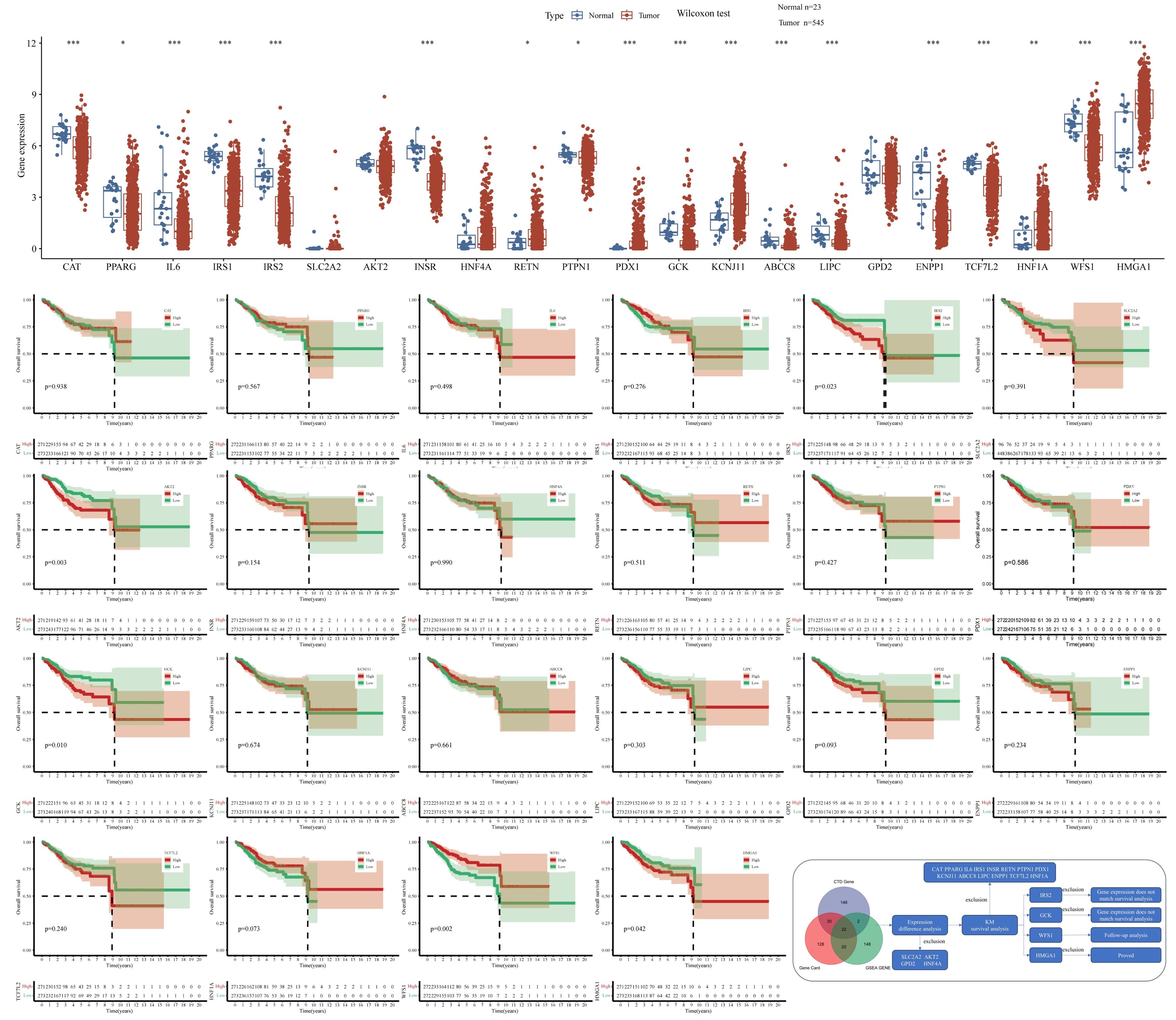

Supplement: Supplementary file 1 [file Image1.tif]
