# Supplementary material for: The immunological and prognostic significance of the diabetes mellitus-related gene WFS1 in endometrial cancer
Source: Front Immunol. 2024 Oct 16;15:1464421. doi: 10.3389/fimmu.2024.1464421 (PMC11521820; doi:10.3389/fimmu.2024.1464421)
Supplement: Supplementary file 4 [file Table3.docx]

| Characteristics | Low WFS1 | High WFS1 | P value |
| --- | --- | --- | --- |
| n | 272 | 273 |  |
| Clinical stage, n (%) |  |  | 0.030 |
| Stage I | 155 (28.4%) | 185 (33.9%) |  |
| Stage II | 27 (5%) | 25 (4.6%) |  |
| Stage III | 70 (12.8%) | 54 (9.9%) |  |
| Stage IV | 20 (3.7%) | 9 (1.7%) |  |
| BMI, n (%) |  |  | 0.166 |
| <= 30 | 111 (21.6%) | 98 (19.1%) |  |
| > 30 | 143 (27.8%) | 162 (31.5%) |  |
| Histologic grade, n (%) |  |  | < 0.001 |
| G1 | 38 (7.1%) | 61 (11.4%) |  |
| G2 | 39 (7.3%) | 82 (15.4%) |  |
| G3 | 187 (35%) | 127 (23.8%) |  |
| Age, n (%) |  |  | 0.005 |
| <= 60 | 87 (16.1%) | 120 (22.1%) |  |
| > 60 | 182 (33.6%) | 153 (28.2%) |  |
| OS event, n (%) |  |  | 0.002 |
| Alive | 213 (39.1%) | 241 (44.2%) |  |
| Dead | 59 (10.8%) | 32 (5.9%) |  |
